# Can smaller predators expand their prey base through killing juveniles? The influence of prey demography and season on prey selection for cheetahs and lions

**DOI:** 10.1007/s00442-023-05335-8

**Published:** 2023-03-04

**Authors:** Eleesha Annear, Liaan Minnie, Kaeleah Andrew, Graham I. H. Kerley

**Affiliations:** 1 Department of Zoology, Centre for African Conservation Ecology, Nelson Mandela University, Gqeberha, 6031 South Africa; 2 School of Biology and Environmental Sciences, University of Mpumalanga, Mbombela, 1200 South Africa

**Keywords:** Demographic-specific predation, Prey preference, Seasonal diet, Lion, Cheetah

## Abstract

Smaller predators may overcome body size restrictions on their prey base by selecting for juveniles of larger prey species. However, traditional prey selection models ignore demographic classes within prey species. We refined these models for two predators with contrasting body sizes and hunting strategies, by including seasonal consumption and availability of prey demographic classes. We predicted that cheetahs would select for smaller neonate and juvenile prey especially of larger species, while lions would select for larger, adult prey. We further predicted seasonal diet shifts in cheetah, but not lion. We recorded species-specific demographic class prey use (kills) via direct observation and GPS cluster of cheetahs and lions fitted with GPS collars. Species-specific demographic class prey availability was estimated from monthly driven transects, and species-specific demographic class prey preferences were estimated. The availability of prey demographic classes varied seasonally. Cheetahs preferred neonates, juveniles, and sub-adults during the wet season, but adults and juveniles during the dry season. Lions preferred adult prey irrespective of season, with sub-adults, juveniles, and neonates killed relative to their abundance. This confirms that traditional prey preference models do not adequately account for demographic-specific prey preference. This is particularly important for smaller predators, like cheetahs, that focus on smaller prey but can expand their prey base by killing juveniles of larger species. For these smaller predators, prey availability will vary strongly seasonally, making them more vulnerable to processes that influence prey reproduction, like global change.

## Introduction

Smaller predators typically kill smaller prey due to body size constraints on their physical ability to capture and subdue prey, and the risks that larger prey may impose (Clements et al. 2016). This limits the prey accessible (sensu Clements et al. 2014) to smaller predators, which is particularly apparent in systems with a diverse range of potential prey species that vary widely in body size, such as the ungulate communities found in African systems. However, body size of potential prey species varies with life history phases, and an obvious adaptive response to the prey size limitations of smaller predators would be for them to exploit the smaller neonates and juveniles of larger prey species (Hayward et al. 2006c; Makin and Kerley 2016). In contrast, larger predators, with their ability to subdue larger prey (Hayward and Kerley 2005) and higher food demands, would be expected to focus on the larger adults of accessible prey species. By extension, the seasonal availability of neonate and/or juvenile prey (Ogutu et al. 2014) should lead to seasonal diet shifts in smaller, but not larger, predators. Here we develop and test this hypothesis by first revisiting the development of prey preference models to account for potential preferences for demographic classes within prey species. We then contrast the demographic-specific prey preferences of a smaller (cheetah *Acinonyx jubatus*), and a larger (lion *Panthera leo*) predator, and explore the implications of seasonal neonate prey availability on these prey preferences.

Prey selection occurs when a predator kills prey species at frequencies higher (preference) or lower (avoidance) than predicted by the relative availability of the prey (e.g., Hayward and Kerley 2005). According to optimal foraging theory (MacArthur and Pianka 1966; Charnov 1976), several factors, particularly prey abundance and vulnerability, influence prey selection by predators. More abundant prey are encountered more often, and all else being equal, will yield a greater energetic return than scarce prey (MacArthur and Pianka 1966; Charnov 1976). Thus, prey availability is determined by the abundance of prey, and their vulnerability (MacArthur and Pianka 1966; Charnov 1976). In areas with seasonal rainfall (i.e., wet and dry seasons), prey species typically give birth during the wet season when there is a high availability of food and water (Ogutu et al. 2014). Given that the relative abundance and vulnerability of demographic classes within an ungulate prey population varies seasonally, the vulnerable neonates and juveniles (Barber-Meyer and Mech 2008) will be relatively more abundant during or just after the parturition period, typically in the wet season, and rare or absent in other seasons.

Optimal foraging theory suggests that predators would select prey that offers the highest energetic benefits with the lowest energetic costs (MacArthur and Pianka 1966; Charnov 1976). This allows us to predict the prey that predators would feed on (e.g., Hayward and Kerley 2005). Prey selection models, relating prey use to prey availability, have been developed using the Jacobs’ Selectivity Index for lions, cheetahs*,* leopards *Panthera pardus*, African wild dogs *Lycaon pictus* and spotted hyenas *Crocuta Crocuta* (Hayward and Kerley 2005; Hayward et al. 2006a, b, c; Clements et al. 2014), among other species. However, these models are relatively crude, lacking demographic-specific prey resolution. Thus, they assume that all individuals of a prey species are equal, and use a standardized prey species mass of three-quarters of mean adult female body mass (Hayward and Kerley 2005). This standardized mass is assumed to represent the average mass of individuals across the demographic classes (adults, sub-adults, juveniles, and neonates) in the population for that prey species (Schaller 1972; Hayward and Kerley 2005). These crude models, using data on prey availability and use, are then applied to estimate prey selection in relation to prey species’ body size (Hayward and Kerley 2005; Clements et al. 2014). Reflecting their small body size, cheetahs are thus estimated to have a crude accessible prey mass range of 14–135 kg (Clements et al. 2014), preferring medium-sized prey like blesbok *Damaliscus pygarus philipsi*, impala *Aepyceros melampus*, and springbok *Antidorcas marsupialis* (Hayward et al. 2006c). Lions are less limited by prey body size and also hunt cooperatively to access larger prey. Thus, the crude accessible prey mass range for lions is 32–632 kg (Clements et al. 2014), and they prefer medium- to large-prey like gemsbok *Oryx gazella*, blue wildebeest *Connochaetus taurinus*, plains zebra *Equus quagga*, and Cape buffalo *Syncerus caffer* (Hayward and Kerley 2005). However, these crude models require refinement, as they do not differentiate how predators use younger, smaller, and more vulnerable individuals versus the larger adults within the prey species.

**Fig. 1:**
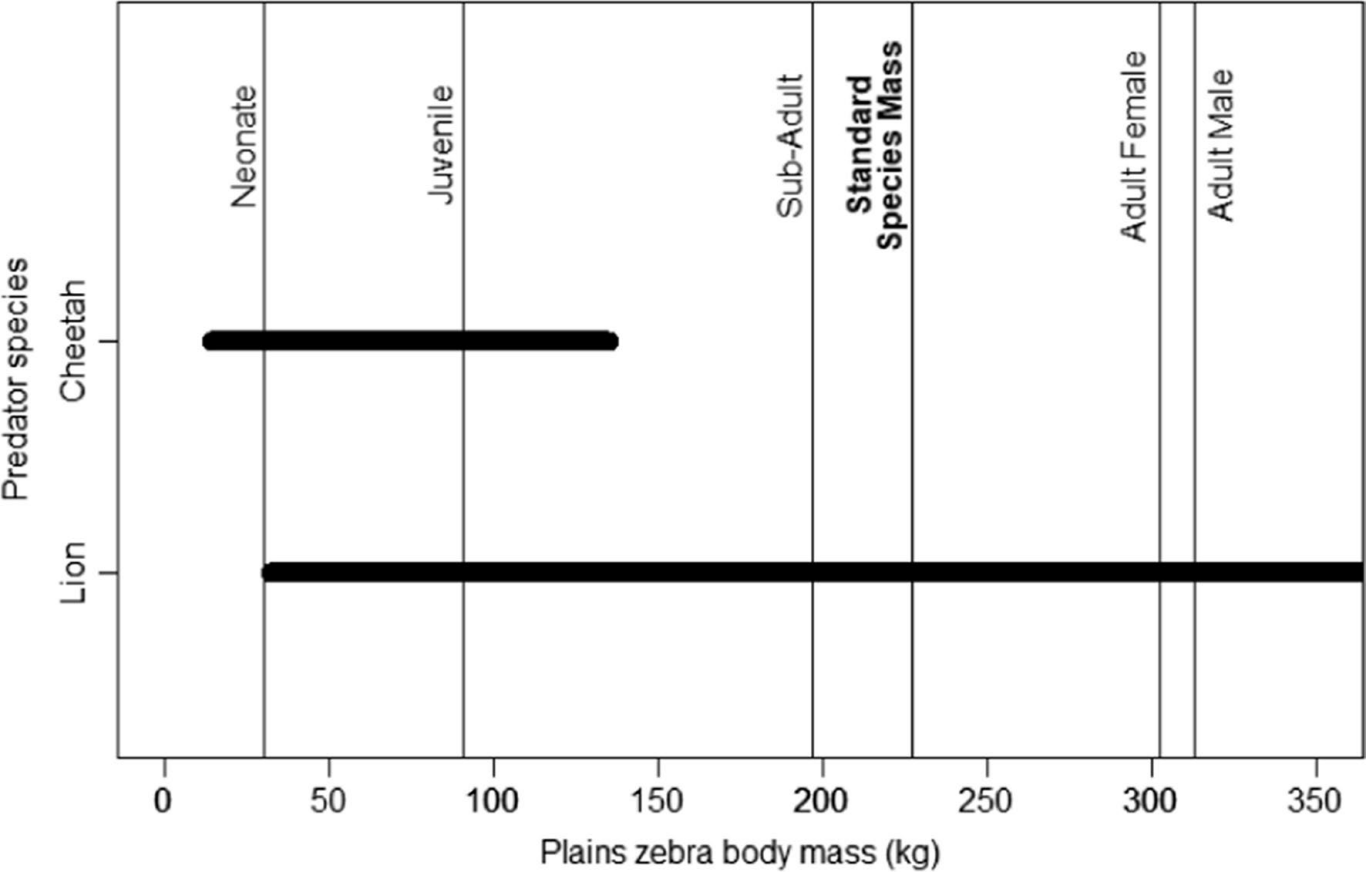
Crude accessible prey mass ranges (horizontal bars) of lion and cheetah plotted across demographic class and standardized masses (vertical lines) of the plains zebra (extracted from Clements et al. 2014; Kingdon et al. 2013)

Ginsberg and Milner-Gulland (1994) found that in over half the studies investigated, predators had a higher percentage of juveniles in their diet than expected. Given the variation in body mass between demographic classes and the seasonal variation in availability (e.g., more neonates after the parturition period) of these demographic classes to predators, prey preferences calculated using the crude models mask demographic-specific prey selectivity, and produce biased predator carrying capacity estimates. For example, plains zebra neonates and juveniles weigh approximately 30 kg and 91 kg, respectively, considerable smaller than adult females (302 kg), adult males (313 kg), and the standardized species mass (227 kg; Fig. 1; Clements et al. 2014; Kingdon et al. 2013). Therefore, using the crude preference model, lions would be expected to prefer plains zebra and cheetahs to avoid plains zebra (Fig. 1; Clements et al. 2014). However, given the crude accessible prey mass range for cheetah, they could hunt plains zebra neonates and juveniles (Fig. 1). Thus, a species-level preference calculation using the standardized species body mass would indicate that cheetah avoids plains zebra, while masking a potential preference for the neonates and juveniles.

Here we refine the crude prey preference models developed by Hayward and Kerley (2005), Hayward et al. (2006c), and Clements et al. (2014) for two predator species differing in body size and hunting strategy—the lion and the cheetah. We hypothesize that prey preference will be influenced by prey demographic class, weaponry, social organization, and season. Due to a relatively smaller body size, cheetahs are restricted to feeding on smaller animals compared to lions (Fig. 1). Therefore, we predict that cheetah will select for neonates and juveniles of large prey species (adult body mass of large prey species: 100–750 kg; Coe et al. 1976), and juveniles, sub-adults, and adults of small prey species (adult body mass of small prey species: 5–90 kg; Coe et al. 1976) during the wet season (i.e., prey parturition period). We also predict that cheetah will select for juveniles of large species and sub-adults and adults of smaller species during the dry season. Furthermore, we predict that cheetahs will prefer prey without defensive weaponry (Clements et al. 2016). In contrast, due to their relatively large body size, we predict that lions will select for adults of larger prey species, irrespective of season and defensive weaponry, but will take adults of smaller prey species, and sub-adults, juveniles, and neonates of larger prey species opportunistically (in proportion to their availability; Barnardo et al. 2020).

## Methods

### Study site

The study was conducted at Lapalala Wilderness Reserve (hereafter Lapalala), a 48 000 ha fenced private reserve (centered at 23°51S, 28°16E) in Limpopo Province, South Africa. The area is located within a summer rainfall region and receives 650–900 mm rainfall per year with the wet season stretching from October to March, and the dry season from April to September (Chizzola et al. 2018). Lapalala is situated within the Lapalala River Basin, on the Waterberg Plateau, a mountainous massif with peaks up to 1 777 masl. The landscape consists of undulating rocky hills with elevated plateaus. The vegetation of Lapalala is characterized as woodlands representative of the Savanna Biome, specifically the Waterberg Moist Mountain Bushveld vegetation type (Rutherford et al. 2006).

Lapalala supports an abundant and diverse large herbivore community, providing a broad prey base for predators. The large herbivore community is dominated by impala, blue wildebeest, plains zebra, warthog *Phacochoerus africanus*, and kudu *Tragelaphus strepsiceros* (Fig. 3). Prior to the reintroduction of lions, cheetah, and spotted hyenas in 2019, Lapalala supported low densities of resident carnivores, including brown hyena *Parahyaena brunnea*, leopard, and black-backed jackal *Canis mesomelas*, in addition to low densities of apparently transient carnivores, including African wild dogs and cheetahs (H. Muller, pers. comm.).

### Cheetah and lion diet

Lions and cheetahs were fitted with GPS collars by reserve management as per Lapalala’s predator management protocols. Data were collected from six adult lions and six adult cheetahs. Lion collars recorded locations every four hours, and cheetah collars recorded locations every two hours. Permission for secondary use of the location data from these animals was granted by the Nelson Mandela University Research Ethics Committee: Animal (A19-SCI-ZOO-006). We assessed diet from kills located at GPS clusters, and through direct observations of the predators.

Potential kill sites were characterized as a cluster of two or more consecutive GPS fix locations within 100 m of each other (Tambling et al. 2010). We inspected clusters 1–14 days after the event to reduce risk to the researchers and disturbance to the predators, and to accommodate field logistics. Clusters were investigated on foot, and we searched a 100 × 100 m area around the center point of a cluster for evidence of a feeding event. A feeding event (i.e., kill site) was characterized by signs of soil disturbance and broken vegetation associated with a struggle, and prey remains, such as gut contents, hair, bone fragments, horns, blood, and a carcass (Davidson et al. 2013). Sites where we did not find any evidence of a kill were classed as resting sites. The remains were used to identify the prey to species level, and where possible, to demographic class. Hair samples were collected where available to aid species identification, using macroscopic characteristics and cuticular scale patterns following van de Ven et al. (2013). The cuticular scale patterns were compared to a hair reference collection at the Centre of African Conservation Ecology (Ott et al. 2007) and published hair reference keys (Keogh 1983; Buys and Keogh 1984). We do not expect the longer collar location intervals for lions to materially influence the evidence for use of smaller prey by lion, as Gerber (2017) showed minor differences in lion diet when comparing 4 h location clusters and simultaneous scat analysis. Furthermore, to back-up the GPS cluster kill data, kills were reported opportunistically by reserve management and tourism staff, and were also located during continuous follows of the predators from August 2019 to July 2020 (Andrew 2022).

### Prey abundance

**Fig. 2:**
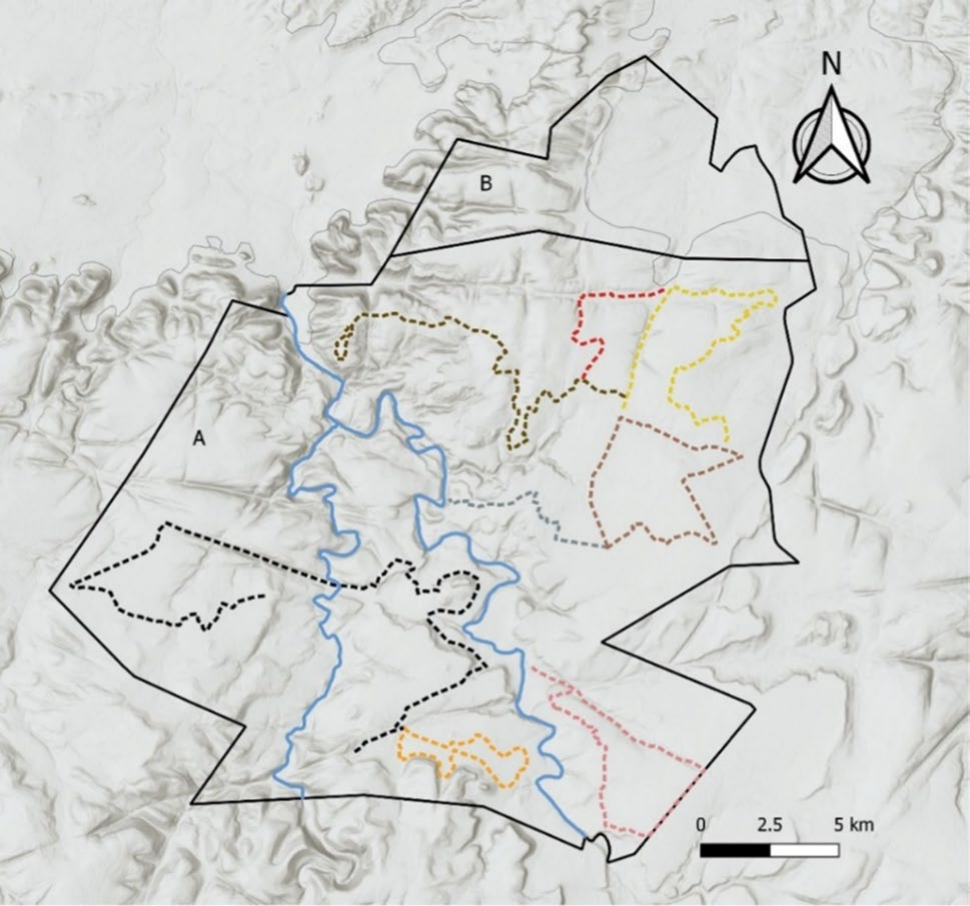
Lapalala Wilderness Reserve showing the eight transects (dotted lines) driven during the monthly prey transects in area A. **A** Southern section of Lapalala with predator and prey species. **B** Northern section of Lapalala with no predators. There is a wildlife-proof fence that separates the northern and southern sections, and the northern section was not used in the present study

Prey abundance in Lapalala was estimated monthly using distance sampling (Buckland et al. 2005) in December 2019–July 2020. This comprised driven transects, totalling 160 km (Fig. 2), selected based on the existing road network, to provide representative coverage of all the habitat types. The transects were driven monthly at < 20 km/hr and data were collected by two observers. Using season, relative body size and horn development, meso-herbivores (medium-sized herbivores ranging in mass from 4 to 500 kg; Coe et al. 1976) and giraffe *Giraffa camelopardalis* were classed into four age classes: neonates were younger than 3 months old, juveniles were 3–12 months old, sub-adults were older than a year, but had not yet reached sexual maturity (i.e., ~ 16 months for all meso-herbivores except plains zebra which mature at ~ 3 years), and adults were individuals that have reached sexual maturity (Skinner and Chimimba 2005; Kingdon et al. 2013). Mega-herbivores (hippopotamus *Hippopotamus amphibius*, elephant *Loxodonta africana*, black rhinoceros *Diceros bicornis*, and white rhinoceros *Ceratotherium simum*), except giraffe, were not included in the prey data as they are rarely preyed upon by lion or cheetah.

Potential prey species were characterized according to body mass, presence of weaponry (i.e., horns, tusks for warthogs and canines for baboons *Papio ursinus*), and average group size. Estimates of the body mass of adult males, adult females, juveniles, and neonates were available for all the prey species (Skinner and Chimimba 2005; Kingdon et al. 2013; Clements et al. 2016). The sub-adult mass was unavailable for most species; thus, this was estimated as the mass halfway between that of adult females and juveniles.

### Statistical analysis

Prey abundance for each demographic class was calculated for the wet and dry seasons, using the program Distance 7.3 (Thomas et al. 2010), calculated using a half-normal key function with a cosine series expansion (Miller and Thomas 2015). Prey abundances and kill data were used to calculate the Jacobs’ Index (JI; Jacobs 1974) per demographic class for each prey species during the wet and dry seasons using:

$$JI= \frac{({r}_{i}- {p}_{i})}{({r}_{i}+ {p}_{i}-2{r}_{i}{p}_{i})}$$

where *p*_*i*_ is the proportion of the prey demographic class *i* that makes up the total abundance of the censused prey and *r*_*i*_ is the proportion of carnivore kills that prey demographic class *i* comprises of. The *JI* ranges from + 1 to − 1, where − 1 indicates maximum avoidance and + 1 indicates maximum preference (Jacobs 1974). The *JI* values were divided into 0.1 interval bins, and each bin was allocated an integer value starting at 1 (bin − 1 ≤ *JI* <  − 0.9) for maximum avoidance and ending at 20 (bin 0.9 ≤ *JI* < 1) for maximum prey preference (Clements et al. 2014). The values ranging from 1 to 20 represent the degree of preference (DOP).

#### Preferred prey mass ranges

Demographic-specific body mass preferences were estimated for the dry and wet seasons for both lions and cheetahs. To ensure non-negative values, the *JI* values were standardized (+ 1; Clements et al. 2014). Segmented generalized linear models (GLM) were used to detect changes in cheetah and lion prey preferences as a function of prey mass during the wet and dry season, following Clements et al. (2014). Prey demographic classes were ordered from lightest to heaviest and ranked with integer values starting from one. The cumulative *JI* values were calculated using successive additions of the standardized *JI* values starting at the species demographic class ranked first (Clements et al. 2014). The breakpoints were then estimated using the segmented GLMs (Gaussian error distribution with a log-link function), by starting with a linear model and incrementally increasing the number of breakpoints detected in each model (Clements et al. 2014). The optimum number of breakpoints was selected using AICc (Akaike’s Information Criterion corrected for small sample size; Symonds and Moussalli 2011). The breakpoints detected in the best fit model were converted back to the corresponding demographic class body masses (Clements et al. 2014).

To determine the significance of preference or avoidance of the prey demographic-class body mass ranges, the mean *JI* value of each prey body mass range, indicated by the different slopes, was calculated. The mean *JI* value was tested for preference, avoidance, or use relative to the species abundance using a single sample *t* test against a mean of zero if data were normally distributed with equal variances. If the mean JI value of the segment slope was significantly less than zero, then the prey with their mass on that segment is avoided. If the mean JI value was significantly greater than zero, then it indicated a preference for that mass range, and if the mean JI value was neither significantly less nor greater than zero, the mass range was used relative to their abundance (Clements et al. 2014).

#### The effect of season and prey characteristics on prey preference

A GLM was used to determine the influence of season, prey demographic class, weaponry, and group size on lion and cheetah prey preference. The DOP for each demographic class was pooled across all prey species. Before running the GLM, binned lion and cheetah DOP were tested for normality using the Shapiro–Wilk test. As both lion (*W* = 0.756, *p* < 0.001) and cheetah (*W* = 0.718, *p* < 0.001) DOP were non-normal, a GLM with a negative binomial distribution and a log-link function was used. The predictor variables were tested for collinearity using a Pearson's chi-squared test. Demographic class and weaponry (*χ*^2^ = 17.56, *p* = 0.003), as well as group size and season *(χ*^2^ = 21.644, *p* = 0.005) were collinear. Thus, prey weaponry and group size were removed from further analyses, as prey demographic class and season were more relevant to our hypotheses and predictions. Model selection was conducted using AICc, and model averaging was used to produce model coefficients from equivalent models (models within two ΔAICc of the top model; Symonds and Moussalli 2011). All statistical analyses were conducted in RStudio (R Core Team 2021).

## Results

### Prey availability

**Fig. 3:**
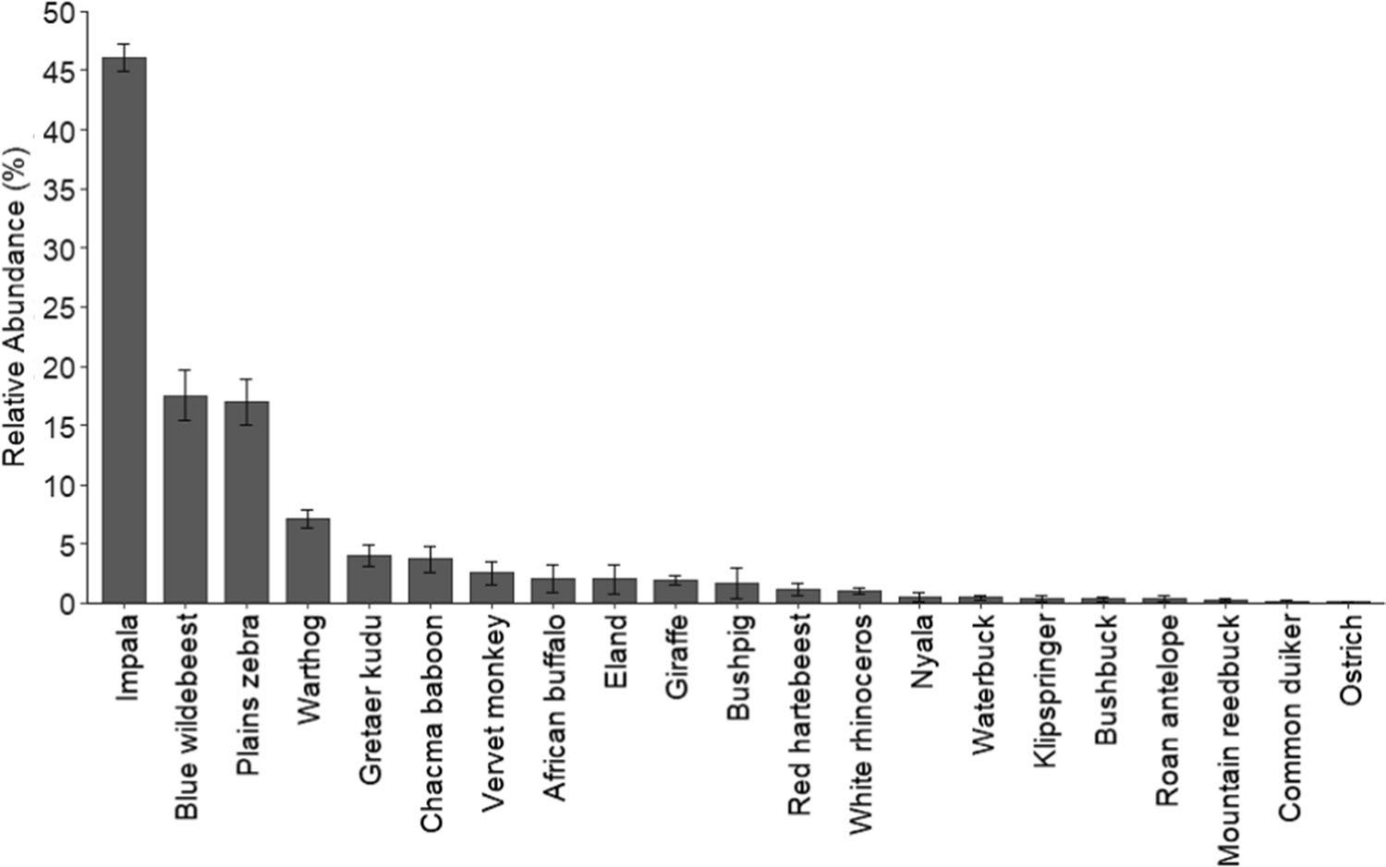
Relative abundance (± SE) of herbivores (pooled across seasons) on Lapalala Wilderness Reserve during 2019–2020

Impalas were the most abundant prey species during the dry and wet seasons (46%), followed by blue wildebeest (17.5%) and plains zebra (17%; Fig. 3). Less abundant prey species included nyala *Tragelaphus angasii*, waterbuck *Kobus ellipsiprymnus*, mountain reedbuck *Redunca fulvorufula*, and common duiker. Ostrich *Struthio camelus* and roan antelope *Hippotragus equinus* were low in abundance as they were released into Lapalala in low numbers during the wet season of this study period.

**Fig. 4:**
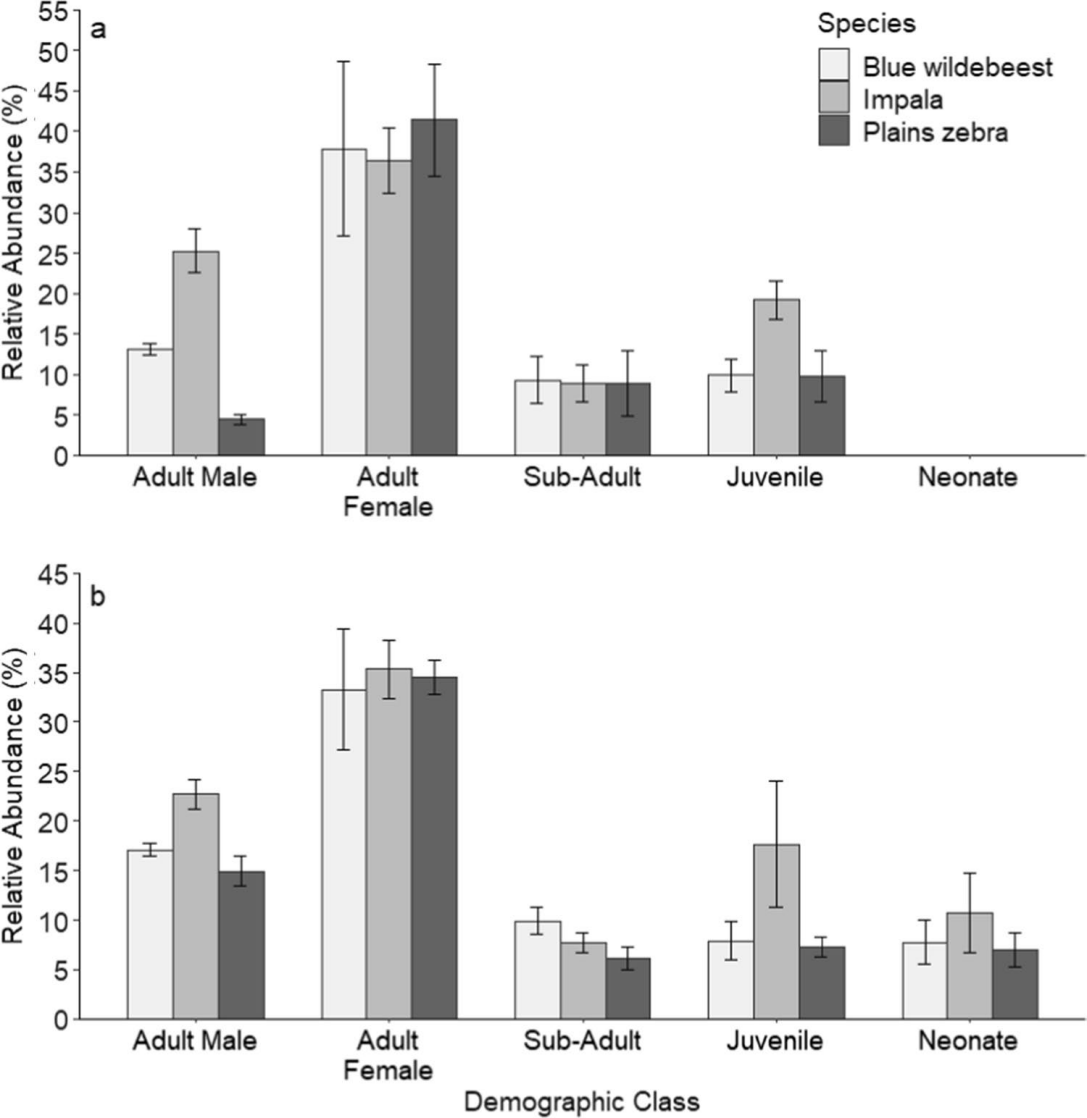
Relative abundance (± SE) of demographic classes of the three most abundant prey species–impala, blue wildebeest, and plains zebra—on Lapalala Wilderness Reserve, during the **a** dry and **b** wet seasons of 2019–2020

The adult demographic class dominated (80%) the prey species on Lapalala. Of the three most abundant prey species - impala, blue wildebeest, and plains zebra - adult females were the most abundant demographic class in the dry (38.5 ± 2.1%) and wet (34.4 ± 0.8%) seasons (Fig. 4). The low abundance of adult male plains zebra during the dry season (Fig. 4a) may reflect the difficulty of identifying the sex of adult plains zebra. Neonates of the three most abundant prey species were absent during the dry season, but present during the wet season (i.e., parturition period; Fig. 4b).

### Prey consumption

We visited 57% (1257 of 2146) of the cheetah and 40% (681 of 1704) of the lion GPS cluster sites, yielding 118 and 147 kill sites for cheetahs and lions, respectively. Direct observations during continuous follows provided an additional 17 cheetah kills and 16 lion kills. In addition, four cheetahs kills, and nine lion kills were recorded through incidental observations by Lapalala staff. Some prey species and/or demographic classes could not be identified, resulting in 85 and 143 identified kills for cheetahs and lions, respectively.

**Fig. 5:**
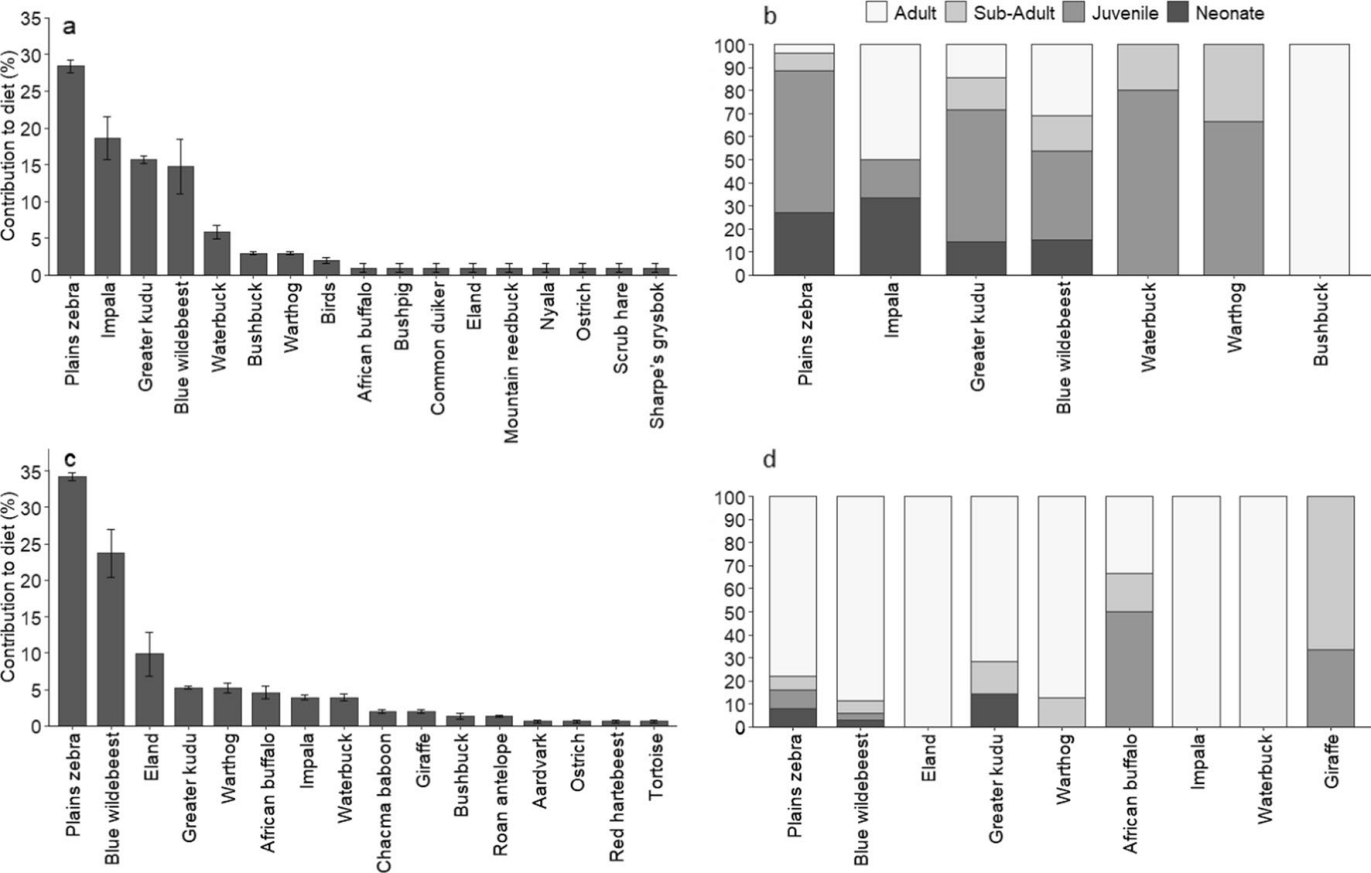
Percentage contribution of prey species, pooled across seasons, to **a** cheetah and **c** lion diet and the demographic composition of prey killed by **b** cheetah and **d** lion on Lapalala Wilderness Reserve in 2019–2020

Cheetah diet was dominated by juvenile and neonate prey (67%) particularly of larger prey species like plains zebra, greater kudu, blue wildebeest, and waterbuck (Fig. 5b). Adult and sub-adult prey only contributed 23% to cheetah diet, with cheetahs killing the adults of smaller prey like impala and bushbuck (Fig. 5b).

Lion diet consisted mainly of adult prey (81%) of large prey species like plains zebra, blue wildebeest, and eland *Tragelaphus oryx* (Fig. 5d). The juvenile prey that lions killed were mostly from larger species like Cape buffalo and giraffe (Fig. 5d). Neonate prey contributed the least to lion diet (Fig. 5d). Plains zebra was the dominant prey species for both cheetahs and lions (Fig. 5a and c). However, cheetahs killed predominantly neonates and juveniles while lions killed adults (Fig. 5b and d). This illustrates the difference in prey demographic class use between a smaller and larger predator. Lions killed neonates the least, these contributing 4% to lion diet overall, with lions killing neonates of large species like greater kudu (14.3% of kudu kills), plains zebra (8% of zebra kills), and blue wildebeest (3% of wildebeest kills; Fig. 5d).

### Preferred prey mass ranges for cheetah and lion

**Fig. 6:**
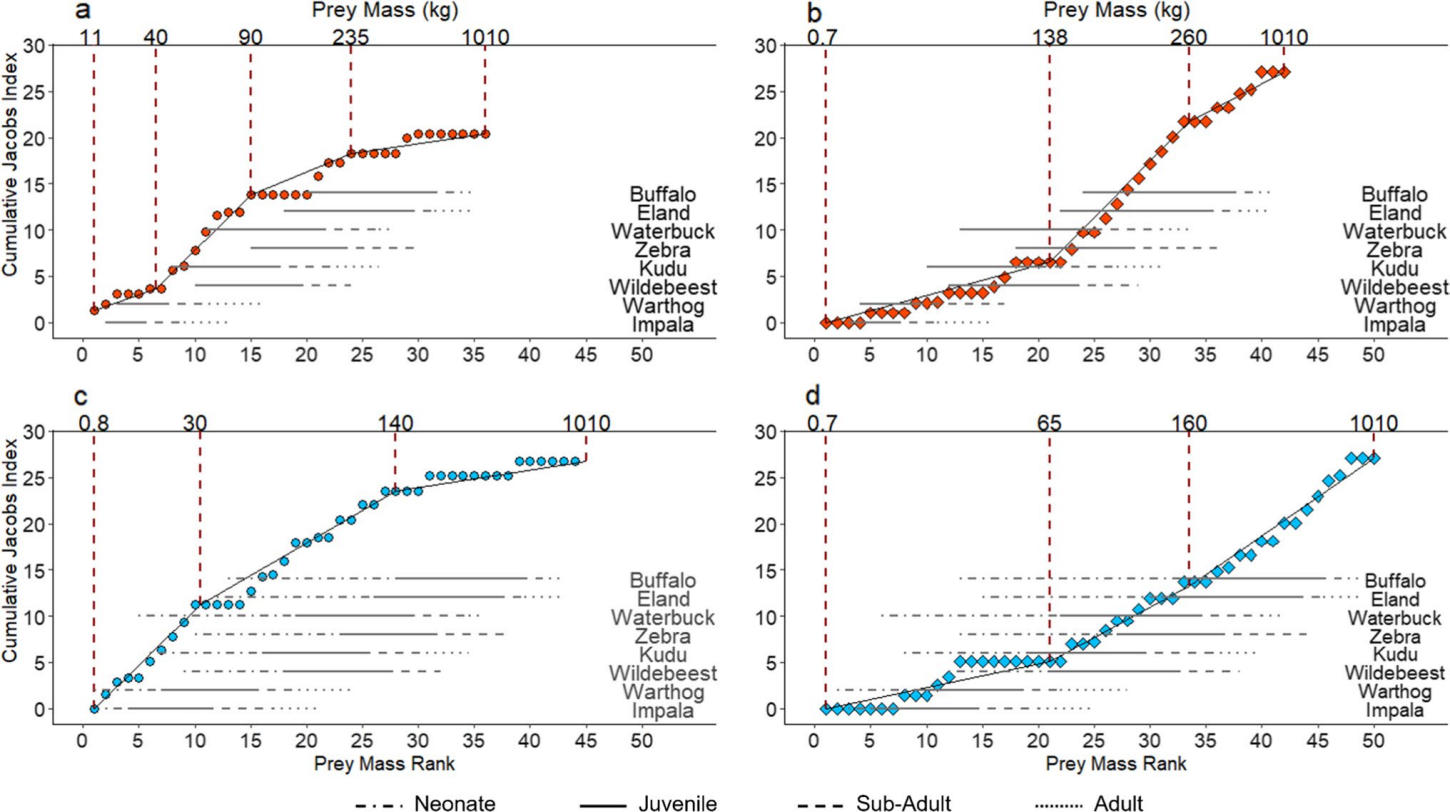
Segmented relationship of cheetah (**a** and **c**) and lion (**b** and **d**) prey preference and the prey mass rank during the dry (orange) and wet (blue) seasons. The seasonally available body mass range (horizontal lines), divided into demographic classes (neonate: mass (kg) from 0 to 3 months old; juvenile: mass from 3 to 12 months old; sub-adult: mass from 12 months to age of sexual maturity; adult: mass from age of sexual maturity), of consumed prey are provided for reference

During the dry season, there were three significant breakpoints in the relationship between prey demographic-class body mass rank and prey preference for cheetah (AICc = 169.09, *n* = 38), corresponding to demographic-specific prey body masses of 40 kg, 90 kg, and 235 kg (Fig. 6a). During the dry season, cheetahs avoided prey classes weighing less than 40 kg (*t* =  − 2.341, *p* = 0.028) and more than 235 kg (*t* =  − 5.649, *p* < 0.001). Prey classes weighing 40—90 kg were preferred (*t* = 4.594, *p* = 0.003); this included adult impala, and juveniles of blue wildebeest, kudu, plains zebra, and waterbuck (Fig. 6a). Prey classes weighing 90–235 kg were consumed relative to their abundance (*t* =  − 1.753, *p* = 0.098), like adult blue wildebeest and kudu (Fig. 6a).

There were two breakpoints in the relationship between prey demographic class body mass rank and cheetah prey preference during the wet season (AICc = 211.73, *n* = 44). These occurred at prey class body masses of 30 kg and 140 kg (Fig. 6b). During the wet season, cheetahs avoided prey classes weighing more than 140 kg (*t* =  − 5.855, *p* < 0.001). Prey classes weighing 0.8—30 kg were preferred (*t* = 5.472, *p* = 0.003), this included neonate impala, blue wildebeest, kudu, and plains zebra (Fig. 6b). Prey classes weighing 30—140 kg, like the juveniles of large prey, were consumed relative to their abundance (*t* =  − 1.624, *p* = 0.123); however, buffalo and eland neonates were avoided. This shows a seasonal shift in prey preferences from adult prey in the dry season to neonate and juvenile prey in the wet season, as predicted.

There were two breakpoints in the relationship between prey demographic class body mass rank and prey preference for lion during the dry season (AICc = 162.27, *n* = 63), corresponding to prey body masses of 138 kg and 260 kg (Fig. 6c). During the dry season, lions avoided prey classes weighing less than 138 kg (*t* =  − 6.521, *p* < 0.001). Lions preferred prey classes weighing 138—260 kg (*t* = 2.619, *p* = 0.013) which included adult and sub-adult waterbuck, plains zebra, kudu, and blue wildebeest, as well as juvenile buffalo (Fig. 6c). Prey classes weighing 260–1010 kg, like adult buffalo and eland, were consumed relative to their abundance (*t* =  − 1.533, *p* = 0.164).

There were two breakpoints in the relationship between prey demographic class body mass rank and lion prey preference during the wet season (AICc = 191.90, *n* = 80). These occurred at prey-class body masses of 65 and 160 kg (Fig. 6d). Prey classes weighing less than 65 kg were avoided by lion (*t* =  − 6.571, *p* < 0.001), and prey classes weighing 65–1010 kg were consumed relative to their abundance during the wet season (*t* =  − 1.633, *p* = 0.128). The breakpoints in lion diet for the wet season occurred at a lower prey demographic class body mass rank than during the dry season (Fig. 6c and d). The lions consumed neonate prey opportunistically, but adult and sub-adult prey continued to contribute the most to lion diet during the wet season. This indicates that, as predicted and unlike cheetahs, lions do not shift their preferences between prey demographic classes seasonally.

### The effect of season and prey demographic class on prey preference

The top model for cheetah prey preference (weight of evidence (*w*) = 42%) included prey demographic class, season, and an interaction between prey demographic class and season (Cheetah model 1, Table 1). Based on the ΔAICc and *w*, the two next best models were also supported (Cheetah model 2: ΔAICc = 0.03, *w* = 41%; Cheetah model 3: ΔAICc = 1.83, *w* = 17%; Table 1). The averaged model indicated that cheetah significantly preferred prey in the juvenile class (model-averaged coefficient = 0.964 ± 0.439, *z* = 2.167, *p* = 0.030), as predicted.

**Table 1:** The top models explaining the variation in the degree of preference (DOP) for prey by cheetahs and lions, based on model selection using Akaike Information Criteria (AICc, Burnham and Anderson 2002)

Model	Description	df	AICc	ΔAICc	Log likelihood	w
Cheetah						
1	DOP ~ DC + S + DC: S	13	510.07	0.00	− 239.57	0.42
2	DOP ~ DC	7	510.10	0.03	− 247.35	0.41
3	DOP ~ DC + S	8	511.92	1.86	− 247.05	0.17
Lion						
1	DOP ~ S	3	607.01	1.45	− 300.38	0.33

*df* Degrees of freedom, *ΔAICc* Difference in relation to the first model, *w* Model weight, *DC* Prey demographic class, *S* Season

The interaction between demographic class and season was significant, with cheetah preferring prey in the neonate (model-averaged coefficient = 3.526 ± 1.469, *z* = 2.361, *p* = 0.018), juvenile (model-averaged coefficient = 1.364 ± 0.644, *z* = 2.085, *p* = 0.037), and sub-adult (model-averaged coefficient = 2.186 ± 0.654, *z* = 3.286, *p* = 0.001) classes significantly more during the wet season than during the dry season, as predicted. This interaction between prey demographic class and season shows a shift in cheetah diet from one dominated by neonate, juvenile and sub-adult prey during the wet season to one dominated by adult prey during the dry season.

Only one best fit model was identified for lion prey preference (Lion model 1, ΔAICc = 1.45; Table 1), which included a single non-significant predictor, season (*w* = 33%; model coefficient =  − 0.185 ± 0.225, *z* = 0.811, *p* = 0.417). This confirms that lions, in contrast to cheetahs, prefer adult prey irrespective of season, as predicted.

## Discussion

Traditionally, prey selection models are based on crude prey-specific body size data, as prey demographic data are typically not collected owing to the high costs and effort of collecting such data (Majumder and Nayak 2015). However, given the potential importance of demographic-specific prey selection, recent research has attempted to refine these crude models via multi-site analyses (Clements et al. 2016). But even these prey selection models do not have appropriate data on availability and use of species-specific demographic classes for predators, thus ultimately reducing our ability to predict the impacts of predation on prey populations. This perspective is particularly important in the context of the hypothesis developed here that smaller predators can expand their prey base by killing juveniles of larger prey species, while larger predators focus on adults of their prey. Both these hypotheses are supported by our data. This study is also a novel attempt to show how predators utilize a diverse prey base in response to seasonal shifts in the abundance of demographic classes. Here, both lion and cheetah prey preferences were influenced, to varying and contrasting degrees, by prey demographic class, and season, as hypothesized. While the findings of this study clearly support the above hypotheses, we do acknowledge that this study was limited to a single site with relatively small populations of both predator species, and look forward to this being tested more broadly.

### Prey demographic class

Prey body size has been demonstrated to be one of the major prey characteristics that influence prey selection (Hayward and Kerley 2005; Khaewphakdee et al. 2020). Body size varies within a species, as between neonates and adults, and between females and males. This demographic variation in prey body sizes has been shown to influence prey selection (Gervasi et al. 2012; Hoy et al. 2015; Heurich et al. 2016; Makin and Kerley 2016), with Pienaar (1969) suggesting over 50 years ago that cheetah specialize in juvenile prey. Furthermore, prey weaponry (horns) is collinear with prey demographic-age class, with neonate and juvenile prey lacking weaponry or at least fully formed weaponry. It has been shown that cheetah prefers prey that lacks weaponry, as this poses a high risk of injury or death for cheetah (Clements et al. 2016; Kerley 2018). Therefore, the preference for juvenile prey by cheetah may also be driven by the lack of defensive weaponry in juveniles, not just their smaller body size.

Lions selected for adult prey, with a small portion of their diet consisted of neonates and juveniles of large species like blue wildebeest, plains zebra, Cape buffalo, and giraffe (Fig. 5). These results are similar to that of Power (2002), who reported that 81% of lion prey were adults. Large, social predators, like lions, prefer adult prey as these offer the most energetic benefit, especially when the kill is shared in a social setting. However, in contrast to what we found, Davidson et al. (2013) indicated that a third of plains zebra kills by lions were juveniles. But Davidson et al. (2013) also emphasized the importance of juvenile mega-herbivores (i.e., giraffe and elephant) in lion diet, particularly during the dry seasons. Although most mega-herbivores, including elephant, hippopotamus, and white and black rhinoceros were not consumed at Lapalala, lions killed one juvenile and one sub-adult giraffe during the dry season.

### Seasonal variation

Predators with a choice of prey species are able to shift prey selection, depending on the relative abundance of prey (Owen-Smith 2008; Corbet and Newsome, 1987). Here we show that such shifts can occur seasonally and at the demographic class level, as per the shift in cheetah diet, from being dominated by adults and juveniles in the dry season, to one dominated by juveniles and neonates in the wet season. This shift is not just in terms of prey category, as it is also expressed by differing accessible prey mass ranges between the dry (40–235 kg; Fig. 6) and wet seasons (0.8–140 kg; Fig. 6). Thus, cheetahs kill larger and older prey over a wider range in prey sizes during the dry season compared to during the wet season, when they kill smaller and younger prey across a narrower range of sizes. This prey switching is supported by the concept of alteration of predation (Corbett and Newsome 1987), which demonstrates how predator diet shifts to enable predators to obtain sufficient resources by foraging optimally over the seasonal cycle. In contrast and as predicted, the accessible prey range for lion did not vary seasonally, this being similar to that estimated by Clements et al. (2014) in a multi-site and –year-scale meta-analysis. The lions’ incorporation of younger demographic classes into their diet during the wet season is a function of their availability, not a preference by lions. This therefore reflects an opportunistic response to the higher abundance of the young demographic classes and is in accord with evidence of opportunistic prey use of lion shown elsewhere (Barnardo et al. 2020).

### Implications of demographic prey selection

Predation is a major driver of prey habitat selection (Brown 1988), population structure, abundance (Paine 1974; Power 2002) and survival (Morin 1985). The impact of predators on prey populations will vary depending on the demographic class selected by predators. For example, predation focused on adults (especially adult females) can influence a prey population by reducing the number of breeding adults and thereby limiting population growth (Gervasi et al. 2012; Hoy et al. 2015). In contrast, predation focused on offspring would reduce the number of offspring that reach sexual maturity, but selective predation on offspring in poor condition may result in a healthier cohort surviving to adulthood (Barber-Meyer and Mech 2008). Furthermore, offspring are produced more rapidly. Thus, a population should be less sensitive to losses of this age class than a higher mortality rate among reproductive-age adults (Linnell et al. 1995; Hoy et al. 2015). The contrasting use of prey demographic classes between large ambush predators and medium-sized pursuit predators shown here indicates that their influences on prey populations may differ. However, when the preferred prey weight ranges of relatively smaller predators, like cheetahs (0.8–90 kg), are nested within the preferred prey weight range of larger predators, like lions (65–1010 kg) (i.e., size-nested predation; Sinclair et al. 2003; le Roux et al. 2019), the synergistic impacts can lead to increased predation pressure on smaller prey (prey species or demographic classes) that is killed by both predators. This may be particularly dire where both adult and non-adult prey of the same species are killed. For example, at Lapalala, cheetahs preferred juvenile blue wildebeest and plains zebra during the dry season and neonate blue wildebeest and plains zebra during the wet season, with lions preferring adult and sub-adult blue wildebeest and plains zebra, irrespective of season. The cumulative predation pressure on both adult and non-adult demographic classes throughout the year may have severe consequences for the persistence of these populations and may result in a shift in prey community structure (le Roux et al. 2019). As such, demographic-specific prey selection plays a major role in shaping prey populations, their demographics and potentially community structure.

Our findings challenge the convention of using the three-quarter of the mean female body mass to estimate prey preference in prey choice studies. The refined model developed here is novel in that it allows prey preferences (and predicted use) to be estimated at a finer, demographic scale. Using the demographic-specific mass instead of the standardized species mass, we were able to highlight how different predators use each demographic class in relation to seasonal changes in availability. The refined models imply that carrying capacity estimates calculated using the crude prey preference models (Hayward et al. 2007) are too coarse for cheetahs and lions. We therefore recommend that these carrying capacity models need to be updated for cheetah based on the demographic-specific prey preferences presented here, and site-specific estimates of cheetah carrying capacity need to be based on prey abundances estimated at the demographic class level. Furthermore, we recommend that adult female body size of prey would be a more accurate predictor of lion prey preference, rather than the three-quarters of the mean adult female body mass.

A further implication of the findings of this study is that processes that influence the reproduction of prey species (i.e., the production and recruitment of neonates and juveniles, and hence their availability as prey) will strongly influence the seasonal prey base and carrying capacity of cheetah (and possibly other smaller predators). This may explain, in part, the general vulnerability of cheetah populations globally, which may be at greater risk of seasonal food limits than larger predators. Global change is characterized by extreme occurrences of drought, fire, flooding, and extreme heat (IPCC 2021). By extension, we speculate that cheetah may be more vulnerable to global change processes than lions, this through these global change impacts on prey reproduction. The lesser impacts predicted for lions reflects the fact that adults are less vulnerable to these extremes than neonates and juveniles (Ogutu et al. 2011). Hence, the lion prey base would be expected to be more stable seasonally. This hypothesis of seasonal bottom-up limitations for cheetah may contribute to a better understanding of why and how this species is threatened and needs to be further explored.

## Conclusion

We provide support for our hypothesis that smaller predators display an adaptive response to prey size limitations by exploiting the smaller (but seasonally available) demographic classes of prey whose larger adults may not be available. The fact that lions, representing larger predators, do not show preferences for neonates and juveniles further supports this hypothesis. Clearly this needs to be further tested through exploring demographic-class preferences by a broader range of predators, and how this may vary in response to seasonal pulses of these demographic classes. Notably, it is important to recognize that these demographic class-specific prey preferences have implications for our understanding of predator–prey interactions and the potential carrying capacity and conservation of predators. This reliance of smaller predators on neonate and juvenile age classes may make them more vulnerable to processes that influence prey reproduction, this at both the shorter term (seasonal) and longer term (global change).

## Data Availability

The datasets generated during and/or analysed during the current study are available from the corresponding author on reasonable request.
